# Do Liquidity Proxies Based on Daily Prices and Quotes Really Measure Liquidity?

**DOI:** 10.3390/e22070783

**Published:** 2020-07-17

**Authors:** Barbara Będowska-Sójka, Krzysztof Echaust

**Affiliations:** 1 Department of Econometrics, Poznań University of Economics and Business, al. Niepodległości 10, 61-875 Poznań, Poland; 2 Department of Operations Research, Poznań University of Economics and Business, al. Niepodległości 10, 61-875 Poznań, Poland; krzysztof.echaust@ue.poznan.pl

**Keywords:** liquidity proxy, liquidity benchmark, volatility estimate, correlation coefficient, partial determination, mutual information

## Abstract

This paper examines whether liquidity proxies based on different daily prices and quotes approximate latent liquidity. We compare percent-cost daily liquidity proxies with liquidity benchmarks as well as with realized variance estimates. Both benchmarks and volatility measures are obtained from high-frequency data. Our results show that liquidity proxies based on high-low-open-close prices are more correlated and display higher mutual information with volatility estimates than with liquidity benchmarks. The only percent-cost proxy that indicates higher dependency with liquidity benchmarks than with volatility estimates is the Closing Quoted Spread based on the last bid and ask quotes within a day. We consider different sampling frequencies for calculating realized variance and liquidity benchmarks, and find that our results are robust to it.

## 1. Introduction

Liquidity is unobservable and elusive concept, which encompasses many transaction properties observed on the markets [1]. Various definitions of liquidity are proposed in the literature related to the bid-ask spreads [2], focused on the price impact of trading volumes [3], or referring to the market depth and dynamics of the order book [4,5]. Liquidity studies are performed on the basis of different information sets with different data frequency and on different markets. In order to maintain a uniform approach we focus on the bid-ask spread as a measure of transaction costs and follow the definition of liquidity as the ability to trade in a reasonable time and at a low cost [6].

Two types of liquidity measures are widely recognized: benchmarks and proxies [7]. In a calculation of benchmarks high-frequency data are required. These data are gathered in big datasets. Dealing with them is highly challenging and time-consuming. In the past decade a number of researchers have sought to determine which liquidity proxy based on low-frequency (daily) data is the best one to represent unobserved liquidity. The competition for the best liquidity proxy relies on the examination of the strength of dependency between proxies and benchmarks [2,7,8,9]. There is no single answer, which measure is the best approximation for the unobserved liquidity and thus its proper measurement is a very demanding process [9,10,11].

Proxies for bid-ask spreads, the so-called percent-cost proxies, are based either on bid and ask quotes (the closing quoted spread of Chung and Zhang [12]) or on high-low-open-close HLOC prices (the effective spread of Corwin and Schultz [8], Abdi and Ranaldo measure [13], or high-low range [14]). The application of the high and low prices is justified by the fact that high prices are usually buyer-initiated prices, while low prices are usually the seller-initiated [8]. However, these prices are also commonly used for non-parametric volatility estimators as e.g., Garman and Klass estimators [15]. Moreover, there is the evidence in the literature that liquidity is related to volatility [16].

To our best knowledge it has not been verified yet, whether daily proxies based on the range of prices and quotes measure unobserved liquidity or volatility. In order to address this gap we examine to what extent liquidity proxies measure liquidity and/or volatility. We employ four benchmarks based on high-frequency data as well as four percent-cost proxies based on daily data [7,17]. Volatility is approximated by two realized variance measures [18] as well as downside and upside realized semivariance [19].

Three approaches are applied: firstly, we investigate the correlation coefficients for proxies and either benchmarks or volatility estimates. Secondly, through the partial determination analysis we examine which of these two, liquidity benchmark or volatility estimate, explains variability of liquidity proxies [20]. Thirdly, we apply mutual information to measure inherent dependencies between any proxy and either liquidity benchmark or volatility estimate [21]. All approaches are conducted within the cross-section and the portfolio time-series settings.

This paper makes a unique contribution to the literature. We find that proxies proposed in the literature based on high-low-open-close prices measure volatility rather than liquidity. The closing quoted spread proposed by Chung and Zhang [12] is the only daily proxy which shows higher dependence with liquidity benchmark than with any volatility estimate. This measure uses the bid and ask quotes observed at the end of the day. Other percent-cost liquidity proxies based on four prices (applied in [8,13,14]) approximate volatility, not liquidity. These conclusions are robust to the changes of an approach undertaken, the cross-section or the portfolio time-series, a method of the dependency measurement and the aggregation of liquidity measures, daily or monthly. They also remain unchanged when high liquidity or low liquidity periods are considered.

Liquidity and volatility are the key factors in price formation process, which are as important in the case of emerging markets as in the case of developed ones. Our study is conducted on the biggest emerging market in the Central and East European countries, on the Warsaw Stock Exchange (WSE). Thus this study extends the understanding of the nature of those relations also on relatively less liquid markets. Our findings are important for both practitioners who seeks for the best liquidity proxies as for academics who deliberate on such measures.

The rest of the paper is organized as follows: Section 2 presents the literature review on volatility and liquidity relation, Section 3 shows the research methodology, Section 4 presents empirical results, Section 5 investigates the robustness of the results in sub-periods and Section 6 concludes.

## 2. Volatility and Liquidity—The Literature Review

Discussion on the relationship between volatility and liquidity has a long history [22,23]. Obviously, these two are of the highest importance to regulators and practitioners. As both volatility and liquidity are latent and both are closely related to the process governing prices, the task of complete distinction of these two is challenging. Karpoff [24] shows the evidence that the large volume and price changes have common sources in the information flow process. Thus the dissemination of information among market participants seems to play crucial role in shaping these two. However, Karpoff did not use a notion of “liquidity”. His seminal paper is on volume, but volume itself might be perceived as a liquidity measure.

The relation between liquidity and volatility in the microstructure theory is not unambiguously defined. In the inventory models this relation is negative [25,26]: higher liquidity implies lower volatility and vice versa. In the information-based models this relation could be also positive [27]: higher liquidity might be accompanied by higher volatility.

The empirical studies show different results in this area. On the one hand Chung and Zhang find that a market uncertainty represented by the Volatility Index, VIX, is a crucial determinant for stock liquidity in the US [12]. Also Ma et al. [28] show that liquidity on the stock markets is lower when investor risk perception reflected by VIX is higher. On the emerging markets Girard et al. [29] find that the relationship between expected volume and volatility is negative and relate it to market inefficiencies. There is the evidence that the transaction costs are higher on the emerging markets [9,30,31]. Also, liquidity tends to decrease when volatility on a domestic market or the market uncertainty measured by VIX increase [32]. On the other hand, Chordia et al. [33] indicate that market volatility induces lower spreads, which means that there is a positive relationship between liquidity and volatility. The evidence from the Chinese stock market is that although market volatility reduces trading activity, it has mixed effects on market liquidity [34].

The difference between the best buy and sell prices, the bid-ask spread, has been historically the most popular measure of liquidity [35]. Domowitz et al. [30] differentiate between liquidity (approximated by trading volume), transaction costs (spreads) and volatility, and consider the relationship between these three variables. They show that higher volatility tends to reduce turnover. In their approach liquidity is separated from transaction costs, while in majority of studies the transaction costs (namely bid-ask spreads) are used to measure liquidity (e.g., [2,25,36,37]).

Summing up, there is a clear distinction between liquidity and volatility in the literature, even if the exact definitions of both concepts vary from one study to another [38,39]. It seems that the liquidity estimates from different dimensions should be interrelated, and they should express stronger dependency with each other than with any volatility estimate.

## 3. Data and Methodology

A vast number of papers is driven by the need of obtaining the best proxy of liquidity at the possible lowest cost. The liquidity measures usually require the access to databases with intraday quotations. The existence of the simple, easy-to-calculate and widely available measure would be appreciated by the market participants. Thus many attempts of creating such a measure on the basis of daily data are made (e.g., [2,7,8,13,36]). We focus on measures based on daily prices or quotations, and examine how strongly are these liquidity proxies related to liquidity benchmarks as well as to well-known volatility estimates.

We consider one market, the Warsaw Stock Exchange (WSE), which is an order-driven market without market makers. This market has been considered as the emerging one [40,41,42,43]. We use long sample of 11 years (2737 days or 133 months); the sample period starts from January 2006 and ends up in December 2016. This 11-year period is long enough to capture different market regimes. Although within this time the WSE was considered as an emerging market, previous studies show that the coherence of liquidity measures is similar to one observed on the developed markets [14]. We take into account quotations of 73 stocks that have been constantly listed within this period and are considered as either big or medium in terms of capitalization. In the case of the WSE it means that they have market value over 50 mln euro. Stocks which experienced splits within sample period were removed from the study. The list of stocks is available upon request. Our primary data come from tick-by-tick database and are cleared from the errors such as multiple records, entries with negative spread, entries for which the spread is more than 50 times the median spread on that day etc. [44]. Finally they are aggregated into equally sampled intraday data.

The empirical framework is conducted on the basis of methodology presented in [7]. Both the cross-section approach for the levels, as well as the portfolio time-series for differences of liquidity measures are applied. The novelty of our approach lies in the examination of interdependence of proxies with benchmarks and with volatility estimates at the same time. Additionally to the calculation of correlation coefficients, we also conduct regression analysis and calculate partial determination coefficients—it allows us to decompose the impact of both benchmarks and volatility measures on variation of proxies. Finally, we examine the dependence between variables using the mutual information measure.

Since the aim of the paper is to examine the relationship between proxies and both benchmarks and volatility estimates, we employ different measures for each of these categories. Starting with proxies we use only percent-cost proxies that are based on the daily values, either *HLOC* prices, or bid and ask quotes. The following proxies are considered:

- effective spread estimator of Corwin and Schultz [8], which rely on the empirical observation, that the highest price within the day *t*, $H_{t}$, is the buyer-initiated price, whereas the lowest price, $L_{t}$, is the seller-initiated price:
  (1) $$CSA_{t}=\frac{2(e^{\alpha_{t}}-1)}{1+e^{\alpha_{t}}},$$
  where $\alpha_{t}=\frac{\sqrt{2\beta_{t}}-\sqrt{\beta_{t}}}{3-2\sqrt{2}}-\sqrt{\frac{\gamma_{t}}{3-2\sqrt{2}}},\beta_{t}={\left(\log\left(\frac{H_{t}}{L_{t}}\right)\right)}^{2}+{\left(\log\left(\frac{H_{t+1}}{L_{t+1}}\right)\right)}^{2}$, and $\gamma_{t}=\log\frac{max(H_{t},H_{t+1})}{min(L_{t},L_{t+1})}$. We adjusted the high-low ratio spread estimator for overnight returns [8];
- the closing percent quoted spread proposed by Chung and Zhang [12]:
  (2) $$CQS_{t}=2\left(P_{A_{t}}-P_{B_{t}}\right)/\left(P_{A_{t}}+P_{B_{t}}\right),$$
  where $P_{A_{t}}$ and $P_{B_{t}}$ are ask and bid quotes, respectively, observed at the end of the day *t*. It is the only one among our proxies that is based on quotes instead of prices;
- the high-low range which is a reformulation of the closing percent quoted spread of Chung and Zhang where the bid and ask quotes are replaced with the high and low prices:
  (3) $$HLR_{t}=2\left(H_{t}-L_{t}\right)/\left(H_{t}+L_{t}\right);$$
- the measure of Abdi and Ranaldo [13] defined as follows:
  (4) $$AR_{t}=2\sqrt{\left(C_{t}-0.5\left(H_{t}+L_{t}\right)\right)\left(C_{t}-0.5\left(H_{t+1}+L_{t+1}\right)\right)},$$
  where $C_{t}$ is the closing price on day *t*.

All daily proxies are interpreted in the same way—the higher the value, the less liquidity is provided.

We also consider different benchmarks, which control for several aspects of the transaction costs. Assume the following notation: there are *K* equally sampled observations within a day, k=0,1,…,K. The benchmarks are defined as follows:

- proportional effective spread
  (5) $$PES_{k}=ES_{k}/MP_{k},$$
  and ES is an effective spread, obtained as
  (6) $$ES_{k}=2D_{k}\left(P_{k}-MP_{k}\right),$$
  where $P_{k}$ is price of the last transaction in an equally spaced time interval (e.g., 5-min), while $MP_{k}$ is the mid price of the best ask quote, $P_{A}$, and the best bid quote, $P_{B}$, within specified interval; $MP_{k}=0.5\left(P_{A_{k}}+P_{B_{k}}\right)$. $D_{k}$ is a variable indicating the direction of the *k*-th trade with 1 and −1 for buy and sell orders, respectively. In order to indicate the direction of a trade, Lee and Ready algorithm is applied [45].
- proportional quoted spread
  (7) $$PQS_{k}=2\left(P_{A_{k}}-P_{B_{k}}\right)/\left(P_{A_{k}}+P_{B_{k}}\right).$$

This spread is based on the quotes only and does not take into account the direction of orders measure [46]. Next two measures are based on the transaction (trade) prices or quotes:

- squared log return on trade prices
  (8) $$SRTP_{k}={\left(\log\left(P_{k}\right)-\log\left(P_{k-1}\right)\right)}^{2},k\neq 0,$$
- midquote squared return
  (9) $$MSR_{k}={\left(\log\left(MP_{k}\right)-\log\left(MP_{k-1}\right)\right)}^{2},k\neq 0.$$

When aggregating over period (day or month) a stock’s liquidity benchmark is calculated as volume-weighted average of its values computed over all *k* observations in the period.

Volatility is approximated by estimates which are based on the logarithmic high-frequency returns:

- realized variance [47]
  (10) $$RV_{t}=\sum_{k=1}^{K}r_{t,k}^{2},$$

where *k* represents an interval and *t* is for a given day. It is assumed that at a sufficiently high frequency and in the absence of jumps, the realized variance can be a good approximation of the unobservable volatility. Thus we also consider minimum RV as a measure that is known to be robust to jumps:

- minRV [18]
  (11) $$minRV_{t}=\frac{\pi }{\pi -2}\frac{K}{K-1}\sum_{k=1}^{K-1}min{\left(|r_{t,k}\left|;\right|r_{t,k+1}|\right)}^{2}.$$

Additionally, we also consider two realized semivariances that allow to focus on the particular risk of long or short position [19]:

- downside realized semivariance
  (12) $$sRVd_{t}=\sum_{k=1}^{K}r_{k}^{2}\times I\left(r_{t,k}<0\right),$$
  where *I* is an indicator variable conditioning calculation of the variance only on the basis of negative returns.
- upside realized semivariance
  (13) $${sRVu}_{t}=\sum_{k=1}^{K}r_{k}^{2}\times I\left(r_{t,k}>0\right),$$
  where *I* is an indicator of positive returns.

All benchmarks and volatility estimates are calculated with the highfrequency R package [48].

## 4. Empirical Research

The empirical research is divided into three parts. Firstly, we apply cross-section analysis for the levels of liquidity and volatility measures as well as portfolio time-series analysis for the first difference of time-series. Secondly, we provide results for the partial determination coefficient analysis, which enables us to differentiate between the relation of a proxy with a liquidity benchmark and volatility estimate. In the last step we calculate mutual information which quantifies the amount of information about a proxy obtained through observing the benchmarks or volatility estimates.

**Figure 1:**
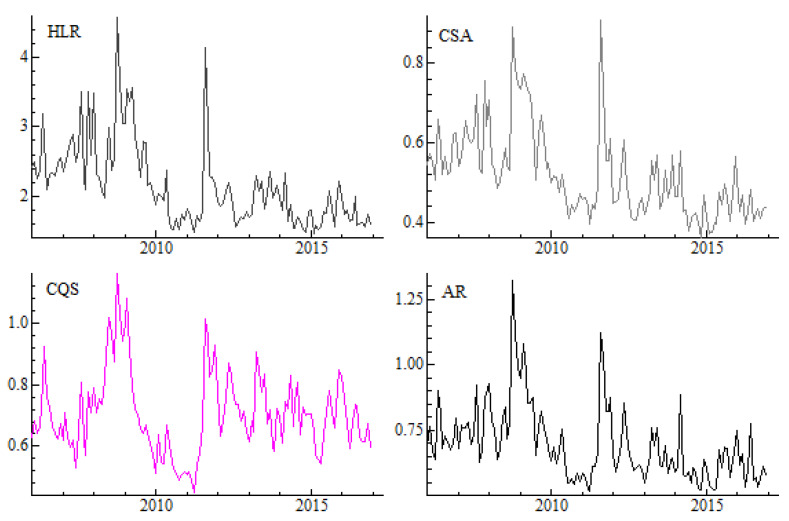
Monthly proxies calculated in the cross-section approach. Note: The following liquidity proxies based on the daily data aggregated to monthly frequency are presented: *HLR* stands for the high-low range [14], *CSA* is spread estimator of [8], *CQS* is the closing quoted spread of [12], while *AR* is the spread estimator of [13]. All graphs are prepared in OxMetrics [49].

Before examining the dependency between considered variables, we present averages of our proxies, benchmarks and volatility estimates aggregated into a monthly frequency. Figure 1 shows that the dynamics of the four proxies based on daily prices and quotes are similar. The average values of proxies are increasing (and liquidity is decreasing) in the time of global financial crisis in 2008 as well as in mid 2011 as a result of the sovereign debt crisis in Europe. This pattern is common across all daily proxies.

**Figure 2:**
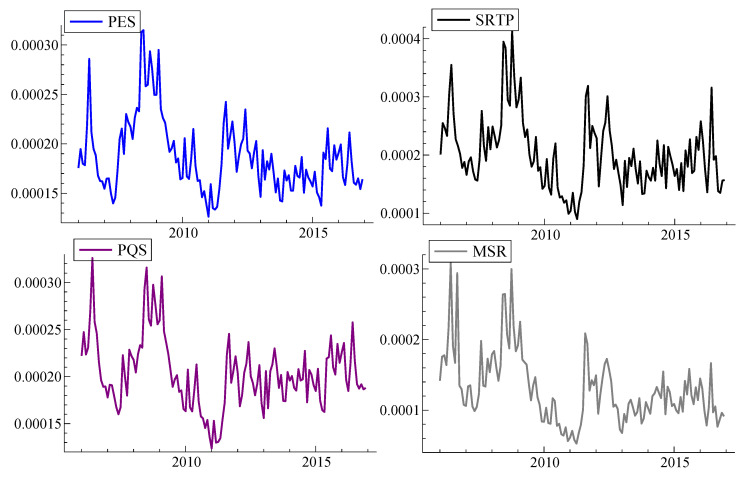
Monthly liquidity benchmarks in the cross-section approach. Note: This graphs shows liquidity benchmarks obtained on the basis of 5-min data and aggregated to monthly values in a cross-section approach. *SRTP* is squared log return on trade prices [48], *PES* is the proportional effective spread [35], *PQS* is the proportional quoted spread [50], and *MSR* is the midquote squared return [48]. For the sake of comparison, *SRTP* and *MSR* are multiplied by 100.

**Figure 3:**
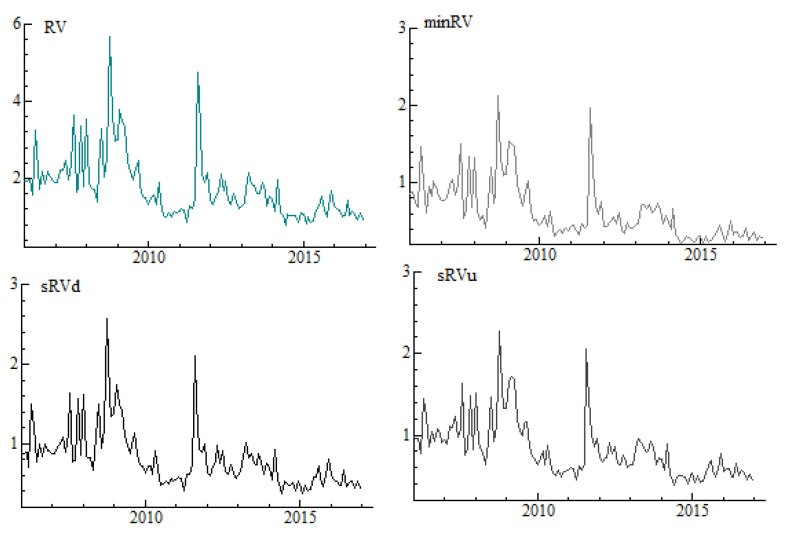
Monthly volatility estimates in the cross-section approach. Note: The following estimators of volatility calculated in 5-min frequency and aggregated to monthly values are considered: *RV* is realized variance [18], *minRV* is minimum realized variance [18], *sRVd* and *sRVu* are downside and upside realized semivariances, respectively [19].

Figure 2 presents liquidity benchmarks considered in the study. We find that the overall trends and comovements of measures are quite similar. The higher the benchmarks’ values, the less liquidity is provided. Finally, the dynamics of four volatility estimates in the cross-section approach is presented in Figure 3. There are no substantial visual differences in behaviour of the series, besides the fact that realized variance, RV, displays the highest values. Both realized semivariances measure risk of either positive returns (upside realized semivariance sRVu) or negative returns (downside realized semivariance sRVd), while minRV is robust to jumps measure of volatility.

### 4.1. Cross-Section Analysis

The average cross-sectional correlations between liquidity proxies and liquidity benchmarks or volatility estimates are computed according to the research methodology presented in [7]: for each day (and each month) we calculate the cross-sectional correlations across all firms in the sample, and then the average correlation is calculated over all days (or months). Spearman rank correlations are applied. This analysis is provided in four different frequencies and with the respect to all considered measures of either liquidity or volatility, for daily and monthly measures separately. We check if the correlations are different between each proxy-benchmark and proxy-volatility pairs using t-test on the time-series of correlations in the spirit of Fama-MacBeth. Following Fong et al. [7], we calculate the cross-sectional correlations and then regress the correlations of one pair on the correlations of another pair. We assume that the time series of correlations of each proxy is IID over time, and examine if the regression intercept is zero and the slope is one. The Newey-West standard errors are applied in order to adjust for autocorrelation [51].

Table 1 presents the Spearman rank ρ coefficients for proxies and liquidity benchmarks calculated on the basis of four frequencies: 5-, 10-, 30-, and 60-minute data, and volatility estimates calculated in the same frequencies. Benchmarks and volatility estimates are presented in pairs for each sampling frequency. Columns 2–5 present the correlations obtained for the series aggregated into daily data, while columns 6–9 display the correlations for series aggregated into monthly data.

For estimates calculated on the basis of 5-min data, we find the evidence that for HLR, CSA and AR correlations with benchmarks are rather low (in absolute values) and definitely weaker than these observed with volatility estimates. It means that proxies based on daily prices (*HLOC*), are closer to volatility estimates than to any benchmark. The mostly striking example is HLR, which is characterized by strong correlations (higher than 55%) with any estimate of volatility, and simultaneously is weakly correlated with benchmarks as PES or PQS. The only opposite case is observed for CQS, for which the correlations with liquidity benchmarks are stronger than with volatility estimates. This finding holds true also when other frequencies are examined.

When focusing on the monthly aggregates, the main result holds: correlations with volatility estimates are stronger than with benchmarks for all proxies, and the only exception is CQS. Our findings show that correlations of CQS with PES and PQS are around 79% and thus are very close to correlation coefficients reported in [7]—79.9% and 91.5%, respectively. For the remaining sampling frequencies similar results are observed. For CQS the lower the frequency of calculating benchmarks or volatility estimates is, the higher the correlation with volatility, but still the correlations with benchmarks remain high.

### 4.2. Portfolio Time-Series Approach

The portfolio time-series approach is based on equally-weighted portfolios across all stocks for a day or a month. We compute a benchmark or volatility estimate in a specified interval by taking the average of detrended benchmarks and volatility estimates over all stocks in a day or a month. The detrending is done by calculating first differences of the time-series. As detrended series are stationary Pearson correlations are calculated. Table 2 shows that independently of the sampling frequency for HLR, CSA and AR the correlations with any estimate of volatility are higher than with any liquidity benchmark. The same situation applies to monthly portfolios.

The remarkable exception among proxies is CQS again, for which in daily portfolios the correlations with both spreads PES and PQS are 47% and 50%, respectively, while the correlations with volatility estimates ranges from 12% to 29% (these numbers apply to the case in which both benchmarks and volatility estimates are based on 5-min data). In monthly portfolios there is no clear answer, which of these two, liquidity benchmarks or volatility estimates, are highly correlated with CQS. As we examine two overlapping correlations with a common variable (proxy), first with a benchmark and second with a volatility estimate, we apply Zou’s test [52]. This test calculates the confidence interval of differences between two correlations. If the confidence interval includes zero the null hypothesis that these correlations are equal cannot be rejected.

We find that considered proxies based on *HLOC* prices are much more related to volatility estimates than to liquidity benchmarks. The only measure which in daily frequency pronounces higher correlation coefficient with two liquidity benchmarks, PES and PQS, is the closing quoted spread, CQS. These results are consistent for all frequencies in which calculation of benchmarks or volatility estimates has been done.

### 4.3. Partial Determination Coefficients

The previous sub-sections are devoted to the correlations between proxies and benchmarks or volatility estimates separately. Here we propose to apply the regression analysis and investigate partial determination coefficients. The idea is the following: we consider linear regression for liquidity and volatility measures, that have been used in the cross-section (in Section 4.1) and the portfolio time-series analysis (in Section 4.2). For the former the equation has a following form:

(14) $$Proxy_{i}=\alpha_{0}+\alpha_{1}\cdot Bench_{i}+\alpha_{2}\cdot RV_{i}+\epsilon_{i},$$

where $Proxy_{i}$ denotes liquidity proxy for stock *i*, $Bench_{i}$ denotes liquidity benchmark, $RV_{i}$ stays for volatility estimate.

For the latter, the portfolio time-series analysis, the equation is following:

(15) $$\Delta Proxy_{t}=\beta_{0}+\beta_{1}\cdot \Delta Bench_{t}+\beta_{2}\cdot \Delta RV_{t}+\epsilon_{t},$$

where Δ is the first difference, and *t* is a time index. The regressions are estimated for both daily and monthly portfolios.

The coefficient of partial determination is the proportion of variation, that can be described by the predictors used in the full model, but cannot be explained in a reduced model [53]. The formula to compute the coefficient of partial determination, $PR^{2}$, is as follows:

(16) $$PR^{2}=\frac{SS_{reduced}-SS_{full}}{SS_{reduced}},$$

where $SS_{reduced}$ is the sum of squares of residuals from the model with only one independent variable, and $SS_{full}$ is the sum of squares of residuals from the full model. In our case the reduced model is a model with either a liquidity benchmark or a volatility estimate, while the full model takes into account both variables simultaneously. Since *RV* among all volatility estimates displays the highest correlation coefficient with proxies, we further show the results for this estimate. As the changes in frequency have no impact on the correlation coefficients and 5-min frequency of observation is usually used as a rule of thumb [54,55,56], henceforth we present results for benchmarks and volatility estimates based on 5-min frequency only (the calculations for remaining proxies and frequencies are available upon request). In calculations rsq R package [57] is applied.

Firstly, we provide results for the cross-section approach. Table 3 presents the determination coefficients, $R^{2}$, as well as partial determination coefficients for both variables, the liquidity benchmark $PR_{Bench}^{2}$ and the volatility estimate $PR_{RV}^{2}$. We find that both for daily and monthly proxies the value of determination coefficient $R^{2}$ varies from 5% to 62%. The comparison of partial determination coefficients, $PR_{Bench}^{2}$ and $PR_{RV}^{2}$, shows that for HLR, CSA and AR both in daily and monthly data partial determination coefficients are much higher for the volatility estimate than for the liquidity benchmark. The only proxy for which we obtain higher partial determination coefficient for liquidity benchmarks than for volatility proxy is CQS. Here in the case of proportional effective spread, PES, in daily data the partial determination coefficient for liquidity is 40% versus 2% for volatility. For PQS the impact of benchmark is 42%, while for volatility it is less than 2% (1.6%). In the case of monthly data, the conclusions are nearly the same.

Secondly, we repeat the procedure for the portfolio time-series approach. Table 4 shows the determination and partial determination coefficients. As in the previous case in daily data CQS is the only proxy for which the partial determination coefficients for liquidity benchmarks, namely PES, PQS and MSR, are higher than for volatility estimates. For monthly data the partial determination coefficients are more balanced: for PES we obtain 30% for liquidity benchmark versus 40% for volatility estimate, while for PQS we get 39% versus 36%. Still CQS seems to represent liquidity, while the other proxies measure volatility.

### 4.4. Mutual Information

So far we have used dependency measures which assume linearity of the relation. As a robustness check we also apply a mutual information which could be considered as a nonparametric dependency measure. Mutual information is an estimate of inherent dependence between two random variables. It specifies the “amount of information” that is shared by two variables and is expressed in terms of the joint probability distribution. The MI concept comes from the Information Theory and is closely related to that of entropy [58,59,60]. The entropy of random variable *X*, H(X), is expressed in the following way:

(17) $$H\left(X\right)=E\left[-\log_{2}\left(p\left(X\right)\right)\right]=-\sum_{i=1}^{L}p\left(x_{i}\right)\log_{2}\left(p\left(x_{i}\right)\right),$$

where p(X) is a probability mass function, while *L* is the length of the time series.

The joint entropy for two random variables, *X* and *Y*, is defined as:

(18) $$H\left(X,Y\right)=-\sum_{i=1}^{L}p\left(x_{i},y_{i}\right)\log_{2}\left(p\left(x_{i},y_{i}\right)\right),$$

where $p(x_{i},y_{i})$ is the joint probability that $X=(x_{i})$ and $Y=(y_{i})$. The mutual information between *X* and *Y* is then defined:

(19) MI(X,Y)=H(X)+H(Y)−H(X,Y).

MI can be then normalized:

(20) $$MI^{*}=MI\left(X,Y\right)/\sqrt{H(X)H(Y)}.$$

The normalized values of $MI^{*}$ are within [0,1] interval, with 0 denoting that both random variables are independent, and 1 denoting they share the same information.

In the study the mutual information measures are calculated both for the cross-section approach and the portfolio time-series approach (we applied the infotheo R package [61]). Table 5 shows the results for the cross-section on daily and monthly data. For proxies versus benchmarks relation in daily data the highest values are obtained for CQS and either PES or PQS. In monthly data all benchmarks have the highest mutual information with CQS. For proxy versus volatility relation, HLR is characterized by the highest mutual information with all volatility estimates both for daily and for monthly data.

Table 6 presents the average mutual information for the portfolio time-series approach. For proxies versus benchmarks the highest mutual information is observed between CQS and PES or PQS. These dependencies are even more pronounced in the case of monthly data.

For volatility versus proxies relation, HLR is featured by the highest amount of mutual information with any volatility estimate, while CQS shows the lowest mutual information with volatility measures. These results hold for both daily and monthly data. Summing up, the results obtained in this Section do not differ significantly from the previous findings.

## 5. Sub-Period Analysis

A potential drawback in our approach is that empirical results may be sensitive to the number of observations taken into considerations. Moreover, some statistical properties may depend on the specifics of the time-series and may be sensitive to the choice of the sample period. This section is devoted to validate the results and assess their consistency. Instead of the whole 11-year period we have chosen two specific two-year sub-periods (484 days) that are closely related to the market liquidity level. The sub-periods are 2007.07.02–2009.06.08. and 2013.01.02–2014.12.30 and relate to the low liquidity and high liquidity regimes, respectively (see Figure 1 and Figure 2). Following previous results we conduct an analysis using all three approaches, the calculation of correlation coefficients, partial determination coefficients and the mutual information for both cross-section and portfolio time-series analyses. Since in the chosen sub-periods there are only 24 months we carry out these computations for the daily aggregation and 5-minute frequency only. The results are shown in the Appendix A in Table A1, Table A2, Table A3, Table A4, Table A5, Table A6.

Generally in sub-periods we find the same relations as in the whole sample. In both periods volatility estimates show higher correlations with proxies than with liquidity benchmarks. The only exception is CQS which demonstrates much higher correlation with benchmarks than with volatility estimates in the cross-section analysis. In the portfolio time-series approach we find high dependence only between CQS and PES or PQS benchmarks. The regression analysis highlights very weak impact of any intraday measures on CSA and AR (low determination coefficient). However, HLR seems to be entirely explained by volatility measures. The mutual information results confirm those obtained in two previous approaches. The highest mutual information is observed for CQS and both PES and PQS. Also we find high mutual information for HLR in association with volatility estimates. These results hold for both sub-periods.

When the comparison between two sub-periods is performed, *CQS* as the best proxy for liquidity indicates higher correlation with *PES* and *PQS* in high liquidity subperiod than in low liquidity time. Hovewer, according to Zou’s test [52] this difference in correlations between high and low liquidity periods are significant only for *CQS-PQS* pair. The regression analysis confirms this finding, i.e., the determination coefficient as a goodness of fit measure is higher in the high liquidity period when the impact of benchmarks in the bivariate relationship is stronger. The mutual information allows to formulate the same conclusions but only in the cross-section approach. In the portfolio time-series the results are ambiguous.

## 6. Conclusions

This paper investigates whether liquidity proxies based on daily data commonly used in the literature indeed approximate latent liquidity. The relations between stock market volatility and liquidity have been the subject of much recent investigation both from the academics’ and practitioners’ point of view. Our research question is driven by the fact that some liquidity proxies, similarly to some volatility measures, apply four prices, that is high-low-open-close prices [15]. In such circumstances, there arises a question, what exactly is measured by a given liquidity proxy. This is an important issue, as there is a need for easy-to-obtain and calculate liquidity measure and many horse races for finding the best proxy are run. The proxies based on range of prices are often examined in such races.

Our results show that measures based on high and low prices capture rather unobserved volatility than liquidity. Both the effective spread estimator of Corwin and Schultz [8] and the spread of Abdi and Ranaldo [13] that have been proposed recently are closer related to different volatility estimates than to any liquidity benchmarks used in the study. Also the high-low range used as a reformulation of the closing quoted spread of Chung and Zhang is less correlated with benchmarks than with volatility estimates. These findings are confirmed by partial determination coefficients from the regression analysis as well as the non-parametric approach based on the mutual information calculation. They hold for the cross-section approach as well as the portfolio time-series.

The only measure based on closing bid and ask quotes, the closing quoted spread of Chung and Zhang [12], has higher dependency with liquidity benchmarks than with volatility estimates. This is confirmed within the correlation analysis, the partial determination coefficients’ analysis and through application of mutual information as a measure of non-linear dependency. All these approaches unanimously indicate that among daily proxies CQS is mostly related to liquidity benchmarks. This proxy is also indicated as the best one in Fong et al. [7].

The answer to the question in the title, “do liquidity proxies based on daily prices and quotes really measure liquidity?” is ‘yes’ for proxies based on daily quotes and ‘no’ for proxies based on daily prices as the latter approximate volatility rather than liquidity. According to our results the proper measurement of liquidity based on daily data requires knowledge of bid and ask prices at the end of the day. Unfortunately this information is not offered in the widely available databases.

## Figures and Tables

**Table 1:** The Spearman rank correlations between liquidity proxies and liquidity benchmarks or volatility estimates—the cross-section analysis.

	Daily				Monthly			
Proxies	HLR	CSA	CQS	AR	HLR	CSA	CQS	AR
Liquidity benchmark: 5 min								
PES	9.13	3.57	66.75 *	10.68	−1.63	−1.45	79.82 *	28.42
PQS	12.15	4.62	68.35 *	10.85	2.92	2.66	78.47 *	29.45
SRTP	40.66	13.53	46.29 *	15.81 ${}^{•}$	18.39	10.87	72.49 *	35.82
MSR	36.62	8.02	37.80 *	9.84	23.70	11.36	66.60 *	34.36
Volatility estimate: 5 min								
RV	73.11	24.38	29.82	19.01	65.90	49.69	52.42	56.19
sRVu	68.07	23.19	25.00	17.17	68.49	52.08	47.27	55.02
sRVd	68.79	25.20	25.93	18.64	66.47	51.53	49.80	55.37
minRV	55.55	19.01	−18.83	6.27	77.69	58.96	−23.75	25.57
Liquidity benchmark: 10 min								
PES	8.30	3.07	66.72 *	10.50	−6.31	−5.05	84.38 *	28.76
PQS	12.26	4.53	68.75 *	8.75	−0.63	0.10	83.70 *	30.48
SRTP	45.53	14.59	42.09 *	15.90	23.03	13.20	67.04 *	36.77
MSR	42.35	9.29	32.01 *	9.83	30.07	14.58	58.15 ${}^{•}$	34.52
Volatility estimate: 10 min								
RV	71.35	23.32	31.09	19.05	60.77	44.70	58.84	56.88
sRVu	65.14	22.00	25.67	17.08	62.48	46.27	55.71	56.34
sRVd	66.04	24.37	27.04	18.83	59.62	45.24	58.81	56.59
minRV	57.49	20.04	−13.60	7.63	78.53	58.52	−9.60	31.27
Liquidity benchmark: 30 min								
PES	8.52	2.92	66.19 *	10.57	−10.22	−8.40	87.67 *	29.18
PQS	13.04	4.89	69.02 *	11.08	−2.94	−1.53	87.33 *	31.32
SRTP	52.85	14.94	34.49 *	15.05	34.85	18.68	54.92	36.19
MSR	50.44	10.22	22.75	9.03	42.66	21.09	41.56	32.33
Volatility estimate: 30 min								
RV	69.17	21.24	30.11	18.52	57.27	38.95	60.69	57.01
sRVu	59.17	19.45	23.47	16.09	57.42	39.34	58.37	56.08
sRVd	60.57	22.79	25.26	18.23	54.28	38.16	61.49	29.18
minRV	58.50	20.34	−6.21	9.88	75.26	55.43	12.56	41.84
Liquidity benchmark: 60 min								
PES	9.25	2.91	64.37 *	10.35	−10.63	−8.93	87.26 *	29.05
PQS	13.21	5.14	68.37 *	11.18	−2.95	−1.16	87.26 *	31.27
SRTP	55.96	14.65	29.53 *	29.53 *	40.89	21.44	48.29	36.59
MSR	54.14	10.61	17.80	8.61	48.79	24.25	34.93	32.79
Volatility estimate: 60 min								
RV	67.93	19.43	27.17	17.45	58.44	36.79	56.43	55.48
sRVu	53.56	17.05	19.76	14.32	57.51	36.59	53.80	53.95
sRVd	55.71	21.32	21.62	17.06	54.52	35.44	56.40	54.03
minRV	55.99	19.37	−4.58	10.67	70.03	50.06	21.26	44.66

Note: The Spearman rank correlations (presented in percentage) are calculated for levels of liquidity proxies and liquidity benchmarks or volatility estimates. The following liquidity proxies are considered: *HLR* stands for the high-low range [14], *CSA* is spread estimator of [8], *CQS* is the closing quoted spread of [12], while *AR* is the spread estimator of [13]. Among liquidity benchmarks *PES* is the proportional effective spread [35], *PQS* is the proportional quoted spread [50], SRTP and MSR are squared return on trade prices and the midquote squared return, respectively [48]. For volatility estimates *RV* is realized variance, [18], *sRVu* and *sRVd* are upside and downside realized semivariances, respectively [19], while *midRV* is minimum realized variance [18]. * signifies that the correlation is statistically significantly higher at the 5% level than the correlation between the same proxy and RV, ${}^{•}$ signifies that the correlation is insignificantly different from the correlation between the same proxy and RV at the 5% level.

**Table 2:** The Pearson correlation coefficients between liquidity proxies and liquidity benchmarks or volatility estimates—the portfolio time-series approach.

	Daily				Monthly			
	HLR	CSA	CQS	AR	HLR	CSA	CQS	AR
Liquidity benchmark: 5 min								
PES	17.53	6.34	47.11 *	3.81	38.25	37.26	63.21 ${}^{•}$	45.23
PQS	20.52	8.87	50.39 *	5.88	51.92	51.92	43.38	44.93
SRTP	18.66	10.32	13.64	5.79	56.65	54.43	70.32 ${}^{•}$	64.88
MSR	6.61	4.60	4.81	1.74	55.62	48.43	62.12 ${}^{•}$	59.38
Volatility estimate: 5 min								
RV	81.42	43.11	24.26	26.51	94.86	84.54	69.40	79.65
sRVu	63.16	27.13	12.10	19.11	94.16	82.52	65.68	76.00
sRVd	78.42	44.45	29.00	25.42	93.97	85.13	69.72	80.46
minRV	73.97	39.41	17.69	24.80	94.28	83.58	62.63	76.33
Liquidity benchmark: 10 min								
PES	18.74	5.34	44.00 *	46.62 *	41.90	40.49	65.24 ${}^{•}$	48.32
PQS	22.10	8.60	49.98 *	6.35	48.24	47.52	75.34 ${}^{•}$	56.27
SRTP	29.55	15.39	14.18	9.65	64.25	59.42	67.27 ${}^{•}$	61.45
MSR	7.80	5.18	3.59	2.30	69.96	61.18	66.27 ${}^{•}$	71.16
Volatility estimate: 10 min								
RV	79.79	42.29	23.56	26.49	94.92	84.08	70.20	79.74
sRVu	53.29	20.67	9.16	15.77	94.22	81.75	66.71	75.56
sRVd	76.29	44.00	28.44	25.23	93.81	84.81	71.43	81.11
minRV	75.46	38.49	20.99	25.55	93.88	83.36	61.45	75.62
Liquidity benchmark: 30 min								
PES	17.64	5.13	48.17 *	2.96	41.75	40.35	69.44 ${}^{•}$	48.39
PQS	21.88	8.31	52.37 *	6.97	47.11	46.73	77.69 ${}^{•}$	54.88
SRTP	29.24	15.99	9.43	8.65	72.02	63.45	58.69	71.57
MSR	17.52	11.89	6.17	6.63	70.95	58.82	57.21	68.03
Volatility estimate: 30 min								
RV	77.23	43.84	21.71	26.91	94.46	82.25	70.08	79.81
sRVu	38.84	13.71	2.81	11.36	93.14	78.39	65.68	73.70
sRVd	72.32	44.42	26.91	24.93	92.91	83.26	71.95	81.78
minRV	74.60	34.40	21.79	24.29	94.43	82.87	64.27	76.83
Liquidity benchmark: 60 min								
PES	17.37	4.72	47.87 *	2.56	40.46	37.54	69.88 ${}^{•}$	45.85
PQS	20.96	8.24	52.59 *	6.63	44.41	44.56	76.86 ${}^{•}$	51.96
SRTP	33.67	17.27	9.37	9.88	68.37	55.49	47.97	63.67
MSR	24.05	13.47	8.20	7.46	67.38	53.70	44.44	62.58
Volatility estimate: 60 min								
RV	76.04	43.39	20.86	26.77	93.99	80.80	69.08	78.43
sRVu	30.77	8.45	−0.87	7.84	91.20	74.61	62.62	69.52
sRVd	69.95	44.39	26.50	25.15	91.97	81.89	71.45	80.95
minRV	72.50	29.13	26.03	20.74	92.99	81.10	64.23	73.32

Note: The Pearson correlation coefficients (presented in percentage) are calculated for differences of liquidity proxies and liquidity benchmarks or volatility estimates. The following liquidity proxies are considered: *HLR* stands for the high-low range [14], *CSA* is spread estimator of [8], *CQS* is the closing quoted spread of [12], while *AR* is the spread estimator of [13]. Among liquidity benchmarks *PES* is the proportional effective spread [35], *PQS* is the proportional quoted spread [50], SRTP and MSR are squared return on trade prices and the midquote squared return, respectively [48]. For volatility estimates *RV* is realized variance, [18], *sRVu* and *sRVd* are upside and downside realized semivariances, respectively [19], while *midRV* is minimum realized variance [18]. * signifies that the correlation is statistically significantly higher at the 5% level than the correlation between the same proxy and RV, ${}^{•}$ signifies that the correlation is insignificantly different from the correlation between the same proxy and RV at the 5% level.

**Table 3:** Partial determination coefficients—The cross-section approach.

	Daily			Monthly		
	$R^{2}$	$PR_{Bench}^{2}$	$PR_{RV}^{2}$	$R^{2}$	$PR_{Bench}^{2}$	$PR_{RV}^{2}$
HLR						
PES	0.586	0.099	0.573	0.620	0.082	0.613
PQS	0.582	0.091	0.566	0.612	0.063	0.596
SRTP	0.569	0.062	0.474	0.602	0.040	0.496
MSR	0.549	0.019	0.466	0.594	0.020	0.485
CSA						
PES	0.096	0.023	0.077	0.082	0.024	0.061
PQS	0.095	0.022	0.076	0.077	0.019	0.057
SRTP	0.093	0.020	0.061	0.076	0.018	0.049
MSR	0.091	0.017	0.070	0.074	0.016	0.053
CQS						
PES	0.465	0.403	0.020	0.393	0.338	0.029
PQS	0.485	0.424	0.016	0.409	0.355	0.016
SRTP	0.241	0.153	0.019	0.202	0.130	0.018
MSR	0.186	0.091	0.035	0.153	0.075	0.025
AR						
PES	0.070	0.020	0.042	0.052	0.020	0.027
PQS	0.070	0.019	0.042	0.051	0.019	0.026
SRTP	0.068	0.017	0.029	0.049	0.017	0.019
MSR	0.065	0.014	0.042	0.048	0.016	0.025

Note: The partial determination coefficients are from linear regression in a form: $Proxy=\alpha_{0}+\alpha_{1}\cdot Bench+\alpha_{2}\cdot RV+\epsilon$ estimated both for daily data (columns 2-4) and monthly data (columns 5-7). Volatility is proxied by the realized variance calculated from the 5-min data. The same frequency is applied to the calculation of different liquidity benchmarks. Among liquidity proxies HLR stands for the high-low range [14], CSA is spread estimator of [8], CQS is the closing quoted spread of [12], while AR is the spread estimator of [13]. Among liquidity benchmarks PES is the proportional effective spread [35], PQS is the proportional quoted spread [50], SRTP and MSR are squared return on trade prices and the midquote squared return, respectively [48].

**Table 4:** Partial determination coefficients—The portfolio time-series approach.

	Daily			Monthly		
	$R^{2}$	$PR_{Bench}^{2}$	$PR_{RV}^{2}$	$R^{2}$	$PR_{Bench}^{2}$	$PR_{RV}^{2}$
HLR						
PES	0.663	0.000	0.652	0.900	0.003	0.883
PQS	0.663	0.001	0.648	0.901	0.007	0.875
SRTP	0.665	0.006	0.653	0.902	0.016	0.855
MSR	0.663	0.001	0.662	0.903	0.027	0.859
CSA						
PES	0.186	0.001	0.183	0.717	0.009	0.672
PQS	0.186	0.000	0.179	0.718	0.013	0.653
SRTP	0.186	0.000	0.177	0.722	0.024	0.604
MSR	0.186	0.000	0.184	0.716	0.004	0.629
CQS						
PES	0.350	0.320	0.014	0.637	0.301	0.396
PQS	0.372	0.343	0.008	0.684	0.390	0.356
SRTP	0.047	0.003	0.036	0.625	0.276	0.258
MSR	0.159	0.120	0.035	0.567	0.165	0.295
AR						
PES	0.071	0.000	0.069	0.659	0.067	0.571
PQS	0.070	0.000	0.067	0.667	0.090	0.544
SRTP	0.071	0.000	0.067	0.693	0.161	0.471
MSR	0.070	0.000	0.070	0.672	0.103	0.494

Note: The partial determination coefficients are from linear regression in a form: $\Delta Proxy=\alpha_{0}+\alpha_{1}\cdot \Delta Bench+\alpha_{2}\cdot \Delta RV+\epsilon$ estimated both for daily data (columns 2–4) and monthly data (columns 5–7). Volatility is proxied by the realized variance calculated from the 5-min data. The same frequency is applied to the calculation of different liquidity benchmarks. Among liquidity proxies HLR stands for the high-low range [14], CSA is spread estimator of [8], CQS is the closing quoted spread of [12], while AR is the spread estimator of [13]. Among liquidity benchmarks PES is the proportional effective spread [35], PQS is the proportional quoted spread [50], SRTP and MSR are squared return on trade prices and the midquote squared return, respectively [48].

**Table 5:** The mutual information—The cross-section approach.

	Daily				Monthly			
	HLR	CSA	CQS	AR	HLR	CSA	CQS	AR
Benchmarks								
PES	5.96	5.51	22.96	5.68	6.76	6.95	35.57	8.21
PQS	6.25	5.55	24.21	5.65	6.71	6.99	35.32	8.49
SRTP	10.82	6.06	13.31	6.33	8.56	7.46	28.27	10.07
MSR	11.28	5.74	10.17	5.82	9.54	7.19	23.27	9.32
Volatility estimates								
RV	26.59	8.14	8.51	7.06	21.78	14.04	16.60	16.27
sRVu	23.52	7.88	8.43	6.61	23.77	14.89	15.03	15.66
sRVd	24.10	8.19	8.63	6.74	22.10	14.34	15.76	16.16
minRV	17.79	7.31	9.25	5.35	30.70	17.33	10.58	7.57

Note: The numbers in table denotes the averages of normalized mutual information (in percentage) between proxies and benchmarks or volatility estimates. The latter two are calculated on the basis of 5-min frequency. The following liquidity proxies are considered: *HLR* stands for the high-low range [14], *CSA* is spread estimator of [8], *CQS* is the closing quoted spread of [12], while *AR* is the spread estimator of [13]. Among liquidity benchmarks *PES* is the proportional effective spread [35], *PQS* is the proportional quoted spread [50], SRTP and MSR are squared return on trade prices and the midquote squared return, respectively [48]. For volatility estimates *RV* is realized variance, [18], *sRVu* and *sRVd* are upside and downside realized semivariances, respectively [19], while *midRV* is minimum realized variance [18].

**Table 6:** The mutual information—the portfolio time-series approach.

	Daily				Monthly			
	HLR	CSA	CQS	AR	HLR	CSA	CQS	AR
Benchmarks								
PES	1.63	1.35	7.48	1.16	6.58	7.14	17.78	10.15
PQS	1.99	1.08	8.54	1.39	8.03	7.47	21.23	11.42
SRTP	2.30	1.49	1.90	1.48	9.20	9.64	18.13	16.25
MSR	2.40	1.70	1.79	1.67	10.79	9.41	17.10	14.16
Volatility estimates								
RV	15.34	3.73	1.99	2.23	31.51	21.95	14.13	20.12
sRVu	8.71	2.48	1.53	1.89	27.70	18.07	12.33	16.62
sRVd	12.21	4.31	2.60	2.75	30.20	25.48	16.15	22.56
minRV	10.00	3.33	1.49	2.21	29.79	22.48	15.12	15.41

Note: The numbers in table denotes normalized mutual information (in percentage) between proxies and benchmarks or volatility estimates. The latter two are calculated on the basis of 5-min frequency. Mutual information is obtained for daily and monthly data separately. The following liquidity proxies are considered: *HLR* stands for the high-low range [14], *CSA* is spread estimator of [8], *CQS* is the closing quoted spread of [12], while *AR* is the spread estimator of [13]. Among liquidity benchmarks *PES* is the proportional effective spread [35], *PQS* is the proportional quoted spread [50], SRTP and MSR are squared return on trade prices and the midquote squared return, respectively [48]. For volatility estimates *RV* is realized variance [18], *sRVu* and *sRVd* are upside and downside realized semivariances, respectively [19], while *midRV* is minimum realized variance [18].

## Appendix A

**Table A1:** The Spearman rank correlations between liquidity proxies and liquidity benchmarks or volatility estimates—The cross-section approach in two subperiods.

	Low Liquidity Period				High Liquidity Period			
	HLR	CSA	CQS	AR	HLR	CSA	CQS	AR
Liquidity benchmark								
PES	6.72	2.96	64.42 *	9.06	12.71	3.41	68.84 *	11.10 ${}^{•}$
PQS	8.39	3.27	65.12 *	8.84	15.64	4.56	71.48 *	11.84 ${}^{•}$
SRTP	37.44	11.28	43.39 *	12.64	42.34	13.78	50.38 *	16.73
MSR	32.44	6.51	37.99 *	7.80	39.45	7.85	41.15 *	10.39
Volatility estimate								
RV	73.00	22.16	26.37	15.88	72.25	24.50	34.77	20.18
sRVu	67.77	21.79	22.50	14.71	67.58	23.43	28.78	18.43
sRVd	69.80	22.08	23.21	14.93	67.71	25.76	29.37	19.58
minRV	59.31	17.69	−16.22	5.03	52.83	19.57	−19.47	7.04

Note: The Spearman rank correlations (presented in percentage) are calculated for levels of liquidity proxies and liquidity benchmarks or volatility estimates. Two periods are taken into account: low liquidity period (2007.07.02–2009.06.08) and high liquidity period (2013.01.02–2014.12.30). For the remaining abbreviations please refer to the notes below Table 1.

**Table A2:** The Pearson correlation coefficients between liquidity proxies and liquidity benchmarks or volatility estimates—The portfolio time-series approach in two subperiods.

	Low Liquidity Period				High Liquidity Period			
	HLR	CSA	CQS	AR	HLR	CSA	CQS	AR
Liquidity benchmark								
PES	13.84	4.46	40.68 ${}^{•}$	1.60	12.53	6.89	44.04 *	8.55
PQS	15.63	7.43	42.94 *	4.02	18.26	12.40	54.76 *	11.67 ${}^{•}$
SRTP	18.18	10.87	7.20	5.95	5.66	4.23	10.33	−2.01
MSR	1.97	2.08	2.87	−1.06	14.17	4.34	8.43	−4.67
Volatility estimate								
RV	81.76	44.57	31.74	25.65	79.51	40.41	26.55	20.74
sRVu	62.54	24.49	19.23	14.07	59.31	16.78	10.97	6.99
sRVd	77.03	46.59	33.48	27.46	77.68	47.40	31.67	25.80
minRV	78.55	40.50	28.34	24.27	68.01	36.20	19.29	21.89

Note: The Pearson rank correlations (presented in percentage) are calculated for levels of liquidity proxies and liquidity benchmarks or volatility estimates. Two periods are taken into account: low liquidity period (2007.07.02–2009.06.08) and high liquidity period (2013.01.02–2014.12.30). For the remaining abbreviations please refer to the notes below Table 2.

**Table A3:** Partial determination coefficients—The cross section approach in subperiods.

	Low Liquidity Period			High Liquidity Period		
	$R^{2}$	$PR_{Bench}^{2}$	$PR_{RV}^{2}$	$R^{2}$	$PR_{Bench}^{2}$	$PR_{RV}^{2}$
HLR						
PES	0.590	0.110	0.578	0.574	0.096	0.558
PQS	0.586	0.102	0.574	0.570	0.088	0.550
SRTP	0.571	0.070	0.492	0.555	0.056	0.449
MSR	0.548	0.021	0.483	0.536	0.017	0.440
CSA						
PES	0.090	0.024	0.071	0.095	0.023	0.077
PQS	0.090	0.023	0.071	0.093	0.022	0.075
SRTP	0.088	0.022	0.059	0.092	0.020	0.060
MSR	0.086	0.019	0.066	0.089	0.017	0.070
CQS						
PES	0.433	0.381	0.016	0.492	0.411	0.023
PQS	0.441	0.390	0.014	0.525	0.449	0.017
SRTP	0.214	0.143	0.018	0.276	0.164	0.016
MSR	0.176	0.101	0.023	0.220	0.099	0.045
AR						
PES	0.062	0.022	0.035	0.070	0.017	0.044
PQS	0.062	0.022	0.035	0.070	0.016	0.042
SRTP	0.060	0.020	0.027	0.069	0.016	0.029
MSR	0.057	0.017	0.034	0.067	0.013	0.043

Note: Two periods are taken into account: low liquidity period (2007.07.02–2009.06.08) and high liquidity period (2013.01.02–2014.12.30). The partial determination coefficients are from linear regression in a form: $Proxy=\alpha_{0}+\alpha_{1}\cdot Bench+\alpha_{2}\cdot RV+\epsilon$ estimated both for low liquidity period (columns 2–4) and high liquidity period (columns 5–7). Volatility is proxied by the realized variance calculated from the 5-min data. The same frequency is applied to the calculation of different liquidity benchmarks. For the remaining abbreviations please refer to the notes below Table 3.

**Table A4:** Partial determination coefficients—The portfolio time-series approach in subperiods.

	Low Liquidity Period			High Liquidity Period		
	$R^{2}$	$PR_{Bench}^{2}$	$PR_{RV}^{2}$	$R^{2}$	$PR_{Bench}^{2}$	$PR_{RV}^{2}$
HLR						
PES	0.669	0.002	0.662	0.637	0.012	0.631
PQS	0.669	0.002	0.661	0.634	0.004	0.621
SRTP	0.673	0.013	0.662	0.633	0.001	0.631
MSR	0.669	0.003	0.669	0.633	0.001	0.625
CSA						
PES	0.199	0.000	0.197	0.164	0.001	0.160
PQS	0.199	0.000	0.194	0.164	0.000	0.150
SRTP	0.199	0.001	0.190	0.163	0.000	0.162
MSR	0.199	0.000	0.198	0.164	0.000	0.162
CQS						
PES	0.235	0.149	0.083	0.221	0.162	0.034
PQS	0.247	0.163	0.077	0.314	0.262	0.021
SRTP	0.101	0.001	0.097	0.077	0.006	0.067
MSR	0.101	0.000	0.100	0.072	0.002	0.066
AR						
PES	0.066	0.000	0.066	0.044	0.001	0.037
PQS	0.066	0.000	0.064	0.047	0.004	0.034
SRTP	0.066	0.000	0.063	0.045	0.002	0.044
MSR	0.066	0.001	0.066	0.049	0.007	0.047

Note: Two periods are taken into account: low liquidity period (2007.07.02–2009.06.08) and high liquidity period (2013.01.02–2014.12.30). The partial determination coefficients are from linear regression in a form: $Proxy=\alpha_{0}+\alpha_{1}\cdot Bench+\alpha_{2}\cdot RV+\epsilon$ estimated both low liquidity period (columns 2–4) and high liquidity period (columns 5–7). Volatility is proxied by the realized variance calculated from the 5-min data. The same frequency is applied to the calculation of different liquidity benchmarks. For the remaining abbreviations please refer to the notes below Table 4.

**Table A5:** The mutual information—The cross-section approach in two subperiods.

	Low Liquidity Period				High Liquidity Period			
	HLR	CSA	CQS	AR	HLR	CSA	CQS	AR
Liquidity benchmark								
PES	6.17	5.33	21.53	5.29	6.38	5.41	24.17	5.90
PQS	6.25	5.36	21.62	5.26	6.84	5.57	26.41	5.81
SRTP	10.37	5.81	12.17	5.83	11.37	6.01	14.83	6.52
MSR	10.54	5.43	10.18	5.51	12.23	5.60	11.13	6.06
Volatility estimate								
RV	26.05	7.61	7.60	6.43	26.38	8.03	9.89	7.28
sRVu	22.98	7.65	7.72	6.37	23.37	7.60	9.68	6.63
sRVd	24.45	7.67	7.68	6.16	23.51	8.22	9.73	6.88
minRV	19.25	7.32	8.72	5.29	16.81	7.16	9.89	5.32

Note: The numbers in table denotes normalized mutual information (in percentage) between proxies and benchmarks or volatility estimates. The latter two are calculated on the basis of 5-min frequency. Two periods are taken into account: low liquidity period (2007.07.02–2009.06.08) and high liquidity period (2013.01.02–2014.12.30). For the remaining abbreviations please refer to the notes below Table 5.

**Table A6:** The mutual information—The portfolio time-series approach in two subperiods.

	Low Liquidity Period				High Liquidity PerioCQSd			
	HLR	CSA	CQS	AR	HLR	CSA	CQS	AR
Liquidity benchmark								
PES	1.95	1.75	11.97	1.02	2.78	2.26	6.21	2.38
PQS	2.77	1.18	12.68	1.75	2.07	3.47	8.13	2.37
SRTP	4.00	1.81	1.80	2.37	2.33	1.86	2.51	2.24
MSR	3.33	2.19	2.64	2.22	3.40	1.65	3.05	2.40
Volatility estimate								
RV	17.50	5.50	2.84	3.38	17.00	3.75	3.84	1.99
sRVu	9.34	3.26	1.77	3.48	11.09	1.61	2.59	1.97
sRVd	12.62	5.72	3.23	3.44	16.27	3.38	4.18	2.26
minRV	14.29	5.23	4.21	3.95	10.77	2.08	3.38	1.89

Note: The numbers in table denotes normalized mutual information (in percentage) between proxies and benchmarks or volatility estimates. The latter two are calculated on the basis of 5-min frequency. Two periods are taken into account: low liquidity period (2007.07.02–2009.06.08) and high liquidity period (2013.01.02–2014.12.30). For the remaining abbreviations please refer to the notes below Table 6.
